# COVID-19 Infection and Medicines in Pregnancy in Canada

**DOI:** 10.1192/j.eurpsy.2024.258

**Published:** 2024-08-27

**Authors:** A. Berard, O. Sheehy, P. Kaul, S. Eltonsy, M. Walker, S. Hawken, S. Bernatsky, M. Pugliese, O. Barrett, A. Savu, R. Dragan

**Affiliations:** ^1^Research Centre, CHU Sainte-Justine , Montreal; ^2^Faculty of Medicine & Dentistry, University of Alberta, Edmonton; ^3^College of Pharmacy, University of Manitoba, Winnipeg; ^4^Ottawa Hospital Research Institute, Ottawa; ^5^Department of Epidemiology, Biostatistics and Occupational Health, McGill University, Montreal; ^6^Department of Epidemiology and Community Medicine, University of Ottawa, Ottawa; ^7^Alberta Health Services, Edmonton; ^8^Manitoba Centre for Health Policy, University of Manitoba, Winnipeg, Canada

## Abstract

**Introduction:**

Although over 100 million pregnant women worldwide are at risk of infection with SARS-CoV-2, little data exists on the impact of COVID-19 and related treatments on maternal/neonatal health.

**Objectives:**

1) To quantify the prevalence of medication use in pregnancy to treat COVID-19; 2) To quantify and compare the risk of adverse pregnancy/neonatal outcomes in those with and without COVID-19.

**Methods:**

In the Canadian Mother-Child population-based cohort (CAMCCO), two key sub-cohorts were identified using prospective data collection of medical services, prescription drugs, hospitalization archives data, and COVID-19 surveillance testing program (02/28/2020-2021). The first cohort included all pregnant women with at least one completed trimester of pregnancy during the study period regardless of pregnancy status (delivery, induced/planned or spontaneous abortion); this cohort was further stratified on COVID-19 status. The second cohort included all non-pregnant women (aged 15-45) with a positive COVID-19 test. COVID-19 infection in pregnant or non-pregnant women was assessed using COVID-19 test results or ICD-10CM codeU07.1 from hospital data. COVID-19 severity was categorized based on hospital admission. Women were considered exposed to COVID-19 medications if they filled at least one prescription for a medicine included in the WHO list in the 30 days pre- or 30 days post-COVID-19 positive test/diagnosis. Considering potential confounders, association between COVID-19 during pregnancy, treated vs not, and perinatal outcomes were quantified using log-binomial regression models.

**Results:**

150,345 pregnant women (3,464 (2.3%) had COVID-19), and 112,073 non-pregnant women with COVID-19 diagnoses were included. Pregnant women with COVID-19 were more likely to have severe infections compared to non-pregnant women with COVID-19 (11.4% vs 1.6%, p< 0.001). The most frequent medications used in pregnancy to treat COVID-19 were antibacterials (13.96%), psychoanaleptics (7.35%), and medicines for obstructive airway disease (3.20%). In pregnancy COVID-19 was associated with spontaneous abortions (adjRR 1.76, 95%CI 1.3, 2.25), gestational diabetes (adjRR 1.52, 95%CI 1.18, 1.97), prematurity (adjRR 1.30, 95%CI 1.01, 1.67), NICU admissions (adjRR 1.32, 95%CI 1.10, 1.59); COVID-19 severity was increasing these risks but COVID-19 treatment with study medications reduced all risks.

**Conclusions:**

Severity of COVID-19 was greater in pregnancy. Antibacterials, psychoanaleptics, and medicines for obstructive airway disease were the most used overall. Severe COVID-19 in pregnancy was associated with higher risks of adverse maternal, and neonatal outcomes.

**Disclosure of Interest:**

None Declared

